# Morphological and Cytophysiological Changes in the Adult Rat Adrenal Medulla after Prenatal and Postnatal Exposure to Endocrine-Disrupting DDT

**DOI:** 10.17691/stm2020.12.2.06

**Published:** 2020

**Authors:** E.P. Timokhina, S.V. Nazimova, D.A. Tsomartova, N.V. Yaglova, S.S. Obernikhin, V.V. Yaglov

**Affiliations:** Researcher, Laboratory of Endocrine System Development, Research Institute of Human Morphology, 3 Tsurupa St., Moscow, 117418, Russia;; Senior Researcher, Laboratory of Endocrine System Development, Research Institute of Human Morphology, 3 Tsurupa St., Moscow, 117418, Russia;; Researcher, Laboratory of Endocrine System Development, Research Institute of Human Morphology, 3 Tsurupa St., Moscow, 117418, Russia;; Head of the Laboratory of Endocrine System Development, Research Institute of Human Morphology, 3 Tsurupa St., Moscow, 117418, Russia;; Senior Researcher, Laboratory of Endocrine System Development, Research Institute of Human Morphology, 3 Tsurupa St., Moscow, 117418, Russia;; Professor, Chief Researcher, Laboratory of Endocrine System Development, Research Institute of Human Morphology, 3 Tsurupa St., Moscow, 117418, Russia

**Keywords:** endocrine disruptors, DDT, adrenal gland, chromaffin cells, the function of the neuroendocrine system, catecholamines, epinephrine, tyrosine hydroxylase

## Abstract

**The aim of the investigation** was to study morphological and cytophysiological changes in the adult rat adrenal medulla after prenatal and postnatal exposure to endocrine-disrupting chemical dichlorodiphenyltrichloroethane (DDT).

**Materials and Methods:**

The experiment was carried out on male Wistar rats (n=30). The rats were exposed to low doses of DDT during prenatal and postnatal development (n=10) and during postnatal development (n=10) only. The average daily intake of DDT by rats was 3.30±0.14 μg/kg. The control (n=10) and experimental animals were sacrificed at the age of 10 weeks (post-puberty), when rat adrenal glands reached their maximum development. Histological samples of the equatorial sections of the adrenal glands were prepared. The adrenal medulla was studied using light microscopy and computer morphometry. Immunohistochemistry was performed to evaluate tyrosine hydroxylase expression in chromaffin cells. Plasma epinephrine content was measured using enzyme-linked immunosorbent assay.

**Results:**

Postnatal exposure of rats to low doses of the endocrine disruptor was found to interfere with adrenal medulla development and cytophysiology of chromaffin cells. Prenatal and postnatal exposure resulted in more profound delay in adrenal medulla development of adult rats as compared to the values of rats exposed postnatally, which was manifested by reduced area of the adrenal medulla and reduced total area of chromaffin cells in its section. Postnatal exposure is manifested by more significant functional disorders including decreased synthesis of tyrosine hydroxylase enzyme in the cytoplasm of chromaffin cells and elevated number of tyrosine-hydroxylase-negative cells in the medulla section, leading to a decrease in plasma epinephrine concentration.

**Conclusion:**

Exposure to low doses of DDT in the early stages of development leads to morphological and cytophysiological changes in the adult rat adrenal medulla and, therefore, reduced functional activity of chromaffin cells, which allows considering the endocrine disruptor DDT a potential risk factor disrupting the function of the neuroendocrine system.

## Introduction

The term “endocrine disruptors” was introduced into science in 1993 to describe chemical compounds disrupting the function of the neuroendocrine system [1, 2]. Dichlorodiphenyltrichloroethane (DDT) is one of the most common neuroendocrine disruptors. Its metabolites are found in living organisms around the globe [3, 4]. Currently, the ability of low doses of DDT to disrupt the production of biogenic amines remains an unexplored aspect of the problem. Studies devoted to it are of particular importance due to increasing number of behavioral disorders and development of behavioral reactions in children and adults, which is attributed by some authors to constant low-dose exposure of the body to various chemicals of anthropogenic origin [2, 5, 6].

We earlier found [7, 8] that prenatal and postnatal exposure of rats to low doses of DDT leads to a decrease in norepinephrine and epinephrine concentration in the systemic circulation. In rats, epinephrine produced by the adrenal glands accounts for more than 80% of its total content in the systemic circulation, and the major proportion of norepinephrine enters the blood from the sympathetic part of the autonomic nervous system [7]. However, the mechanism of biogenic amine synthesis disruption remains unclear.

**The aim of the investigation** was to study morphological and cytophysiological changes in the adult rat adrenal medulla after prenatal and postnatal exposure to endocrine-disrupting DDT.

## Materials and Methods

The study was carried out on male Wistar rats (n=30). The rats were exposed to low doses of DDT during prenatal and postnatal development (experimental group 1, n=10) and during postnatal development only (experimental group 2, n=10).

Immediately after mating with males, female rats of experimental group 1 started to consume aqueous solution of o,p-DDT with the concentration of 20 μg/L instead of water (Sigma, USA). During the pre-weaning period, rat offspring of this group received low doses of DDT with mother’s milk. Upon reaching the age of three weeks, these offspring were transferred to independent feeding and their drinking water was replaced with similar DDT solution. Experimental group 2 included rats that consumed DDT only in the postnatal period from postnatal day 1: first with mother’s milk, and then independently.

The average daily independent consumption of DDT by rats was 3.30±0.14 μg/kg body weight. The consumed dose of DDT was calculated according to the requirements for defining low doses for DDT and the Russian standards for this disruptor content in food [9].

Animals of the control group (n=10) were given tap water. The absence of DDT, its metabolites, and related organic chlorine compounds in water and food was confirmed by gas-liquid chromatography. Rats in the control and experimental groups were sacrificed after the experiment with an overdose of Zoletil at the age of 10 weeks (post-puberty) when the adrenal glands reached their maximum development [10].

The adrenal glands were fixed in Buen’s solution; after histologic diagnosis, equatorial paraffin-embedded sections were prepared and stained with hematoxylin and eosin. The area of the medulla, the total area of chromaffin cells in it and their sizes were measured. The presence of tyrosine hydroxylase (TH) was detected by the immunohistochemical method using rabbit polyclonal antibodies (Abcam, UK). The reaction was visualized using a commercial test system (Abcam). Sections were stained with Mayer’s hematoxylin. Percentage of TH-positive and TH-negative chromaffin cells was counted in medulla sections. The proportion of cells with diffuse and local distribution of the enzyme in the cytoplasm was found among TH-positive cells using microscopy of immunopositive cells with 1000-fold magnification and morphometric measurement of immunopositive site areas. Diffuse distribution was understood as the presence of TH in 90–100% of the area of chromaffin cell cytoplasm, while local distribution implied less than 90% of that area.

Histological preparations of the adrenal glands were examined by light-optical microscopy and computer morphometry using ImageScope software and Leica DM2500 light microscope (Leica Microsystems, Germany).

Blood plasma was obtained by adding ethylenediaminetetraacetic acid (EDTA). Plasma epinephrine concentration was assessed by enzyme immunoassay using a highly sensitive CatCombi reagent kit (IBL International, Germany) with the minimum detectable epinephrine content of 0.03 nmol/L.

Care and use of experimental animals complied with the international guidelines set forth in the Guide for the Care and Use of Laboratory Animals (National Research Council, 2011), and the ethical principles established by the European Convention for the Protection of Vertebrate Animals Used for Experimental and Other Scientific Purposes (Strasburg, 2006). The work was approved by the Ethics Committee of the Research Institute of Human Morphology.

**Statistical data processing** was performed using the Statistica v. 7.0 software package (Statsoft Inc., USA). For quantitative parameters, the correspondence of parameter distribution type to normal distribution law was analyzed using Lilliefors test, Shapiro–Wilk test. Central trends and dispersion of quantitative parameters with approximately normal distribution were presented as the mean value and standard error of the mean (M±m). Independent samples were compared based on quantitative parameters using Student’s t-test allowing for the values of Levene’s test for equality of variance. To compare the samples based on qualitative parameters, χ^2^ was used. The differences were considered statistically significant at p<0.01.

## Results

**Figure 1:**
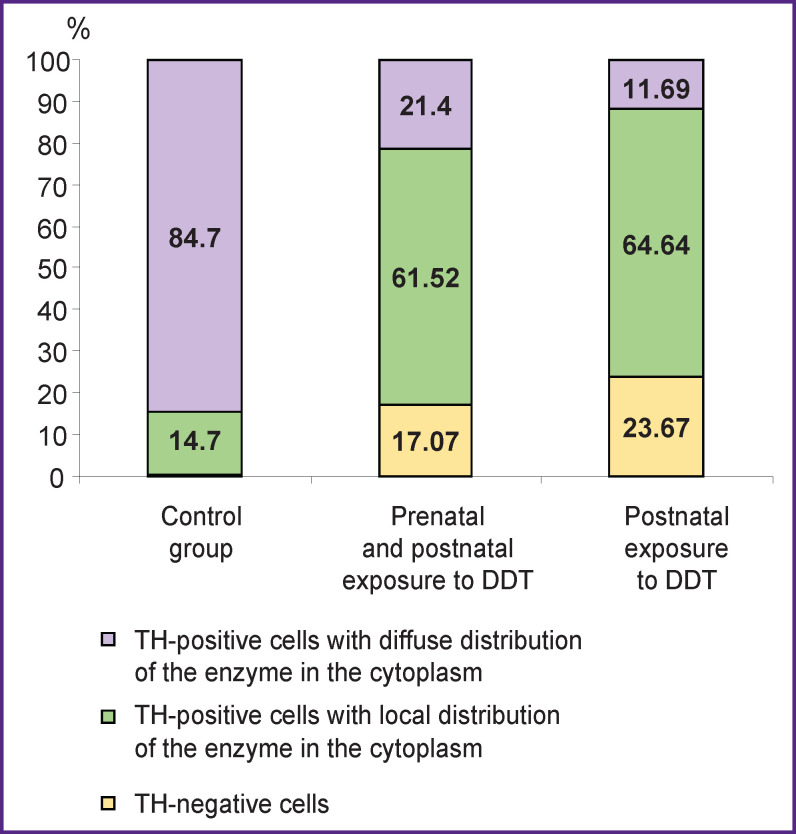
The number of TH-positive and TH-negative cells in the adrenal medulla of rats under study

**Figure 2:**
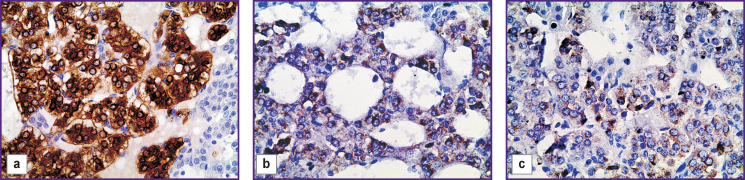
Immunohistochemical evaluation of TH in chromaffin cells of rat adrenal medulla: (a) in the control group (most chromaffin cells have high TH content in the cytoplasm); (b) in rats exposed to low doses of DDT in the prenatal and postnatal periods (decrease in TH content in chromaffin cell cytoplasm); (c) exposure only in the postnatal period (most chromaffin cells do not contain TH); Mayer’s hematoxylin staining; ×400

Rats in the control group had adrenal glands with a typical structure. The cortex and medulla were clearly distinguished on histological sections. Accumulations of chromaffin cells were detected in the medulla, their area was more than two thirds of the total area of the medulla (see the Table). Chromaffin cells had large round nuclei and moderately basophilic cytoplasm. Most chromaffin cells exhibited intense immunohistochemical reaction to TH with diffuse distribution of the enzyme in the cytoplasm. TH-positive cells with local distribution of the enzyme in the cytoplasm were much rarer. Also, single TH-negative cells were found (Figure 1, Figure 2 (a)).

**Table:** Morphological parameters of the adrenal glands of mature rats in the control group and rats exposed to low doses of DDT at various stages of ontogenesis (M±m)

Parameters	control group	Prenatal and postnatal exposure to DDT	Postnatal exposure to DDT
The area of the medulla (μm^2^)	812,000.00±8315.22	598,200.00±45,313.12*	757,000.00±41,000.00^#^
The total area of chromaffin cells in the medulla section (μm^2^)	578,850.00±21,767.91	394,938.00±46,496.07*	544,700.00±19,100.00^#^
The area of the chromaffin cell (μm^2^)	108.39±1.97	112.32±4.40	99.99±3.25*^#^

* Statistically significant differences from the values of the control group; ^#^ differences between the group with “postnatal exposure to DDT” and the group with “prenatal and postnatal exposure to DDT”.

**Figure 3:**
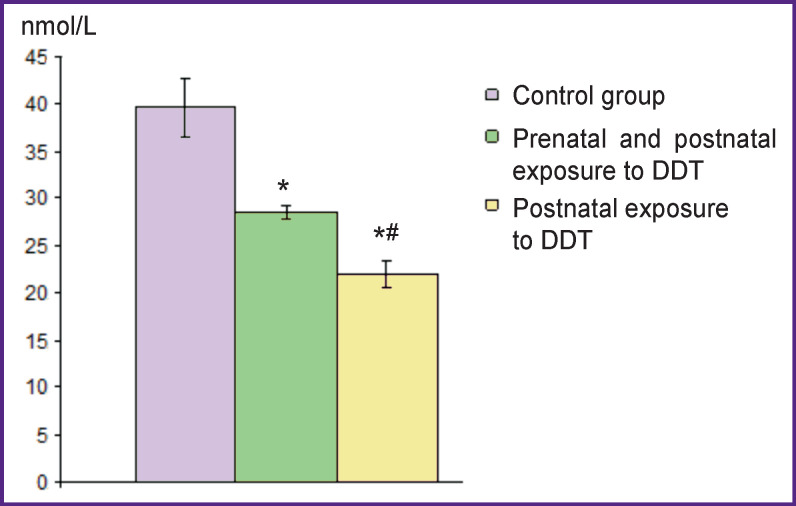
Epinephrine concentration in the blood plasma of the control and exposed rats * Statistically significant differences from the values of the control group; ^#^ from values of the group with “prenatal and postnatal exposure to DDT”

The adrenal glands of rats exposed to low doses of DDT in the prenatal and postnatal periods of ontogenesis also had a typical structure. However, there was a decrease in the medulla area compared to the control group. The area of chromaffin cell accumulations in the medulla section was statistically significantly less than the control values. Comparison of chromaffin cell sizes in this group and the control revealed no differences (see the Table). The number of TH-positive cells was noted to decrease (see Figure 1, Figure 2 (b)), most of them had local distribution of the enzyme in the cytoplasm. The number of TH-negative chromaffinocytes increased statistically significantly. Epinephrine concentration in blood plasma was one-third less than this value in rats of the control group (Figure 3).

The adrenal glands of animals exposed to low doses of DDT only in the postnatal period had a typical structure too. The area of the medulla was statistically significantly larger than that in experimental group 1, and it did not differ from the control values. The total area of chromaffin cells in the medulla section was similar to the control parameters and larger than in the group of rats exposed to DDT in the prenatal and postnatal periods of ontogenesis. At the same time, the size of chromaffin cells as such was reduced as compared to the cells of both the control and experimental group 1 (see the Table). The number of TH-positive cells was the minimum among all the animals studied (see Figure 1). TH was not detected in most of chromaffin cells (Figure 2 (c)). A significant decrease in plasma epinephrine concentration was established as compared to both the control an experimental group 1 (see Figure 3).

## Discussion

The rat adrenal medulla is known to continue developing after the birth of the animal, reaching its maximum development by post-pubertal age [10, 11]. The obtained results show that prenatal and postnatal exposure to low doses of DDT negatively affects the development and functional activity of the adrenal medulla in the post-pubertal period.

In rats exposed to low doses of DDT in the prenatal and postnatal periods of ontogenesis, a decrease in medulla size and the total area of chromaffin cells in the medulla section was revealed after reaching puberty (at the age when adrenal growth normally comes to end [10]), the size of these cells remaining unchanged. This fact implies a decrease in the number of chromaffin cells and delay in the development of the medulla as a whole. The decrease in the development of chromaffin tissue in the adrenal glands of rats exposed to a disruptor was proportional to the decrease in epinephrine production. To determine the mechanism of catecholamine synthesis disruption, quantitative measurement of TH content in chromaffin cell cytoplasm was carried out. Since the data available in the literature suggest that there is only one highly specific reaction in the process of catecholamine biosynthesis — tyrosine hydroxylation to form L-DOPA catalyzed by TH — its intensity can be used to estimate the intensity of catecholamine synthesis [12, 13].

We observed a significant decrease in TH content in chromaffin cell cytoplasm. Since high TH content in the chromaffin cell is a sign of high differentiation, these facts suggest that the decrease in hormone production is associated with a slowdown in the rate of morphogenetic processes due to the action of the endocrine disruptor largely in the prenatal period. Moreover, in the group exposed to low doses of DDT only in the postnatal stage, there was no such pronounced decrease in the size of the medulla compared to the control.

In rats exposed to low doses of DDT only in the postnatal stage of ontogenesis, a decrease in the size of chromaffinocytes set against the unchanged area occupied by these cells in the medulla indicates a compensatory increase in their number in this experimental group. However, a decrease in catecholamine secretion and increase in the number of TH-negative cells were significantly more pronounced than in the animals exposed to low doses of DDT in the prenatal and postnatal periods. This may indicate the developing compensatory processes of reactivation of chromaffin cell secretion due to shorter DDT consumption, manifesting as increased number of functionally immature cells.

The scientific literature provides data on nervous system disorders, mental retardation, and behavioral abnormalities associated with permanent low-dose exposure of the body to various endocrine disruptors [2, 5, 6]. As a rule, changes in mental development are more often associated with the disruptive effect on the thyroid gland function, with impaired secretion of thyroid hormones [14, 15], and impaired steroidogenic activity of genital glands [16, 17]. The results of our study suggest that such disorders may also be associated with the impact of DDT on chromaffin cells, inhibiting catecholamine secretion.

## Conclusion

Developmental exposure of rats to low doses of endocrine-disrupting DDT impairs the development of adrenal medulla and cytophysiology of chromaffin cells. Exposure initiated in the prenatal period is manifested mainly by morphological changes in the medulla after puberty, while exposure in the postnatal period reveals itself in more pronounced impairment of chromaffin cell functioning. Therefore, exposure to low doses of DDT can be considered a potential risk factor disrupting the function of the neuroendocrine system.
